# In-Network Data Aggregation for Ad Hoc Clustered Cognitive Radio Wireless Sensor Network

**DOI:** 10.3390/s21206741

**Published:** 2021-10-11

**Authors:** Mohamad Rida Mortada, Abbass Nasser, Ali Mansour, Koffi-Clément Yao

**Affiliations:** 1 LABSTICC UMR CNRS 6285, ENSTA-Bretagne, 29806 Brest, France; mohamad.mortada@ensta-bretagne.org (M.R.M.); ali.mansour@ensta-bretagne.fr (A.M.); 2 ICCS-Lab, Computer Science Department, American University of Culture and Education, Beirut 1507, Lebanon; 3 LABSTICC UMR CNRS 6285, Université de Bretagne Occidentale, 29238 Brest, France; koffi-clement.yao@univ-brest.fr

**Keywords:** cognitive radio, wireless sensor network, in-network data aggregation, network lifespan, multihop routing

## Abstract

In cognitive radio wireless sensor networks (CRSN), the nodes act as secondary users. Therefore, they can access a channel whenever its primary user (PU) is absent. Thus, the nodes are assumed to be equipped with a spectrum sensing (SS) module to monitor the PU activity. In this manuscript, we focus on a clustered CRSN, where the cluster head (CH) performs SS, gathers the data, and sends it toward a central base station by adopting an ad hoc topology with in-network data aggregation (IDA) capability. In such networks, when the number of clusters increases, the consumed energy by the data transmission decreases, while the total consumed energy of SS increases, since more CHs need to perform SS before transmitting. The effect of IDA on CRSN performance is investigated in this manuscript. To select the best number of clusters, a study is derived aiming to extend the network lifespan, taking the SS requirements, the IDA effect, and the energy consumed by both SS and transmission into consideration. Furthermore, the collision rate between primary and secondary transmissions and the network latency are theoretically derived. Numerical results corroborate the efficiency of IDA to extend the network lifespan and minimize both the collision rate and the network latency.

## 1. Introduction

Currently, wireless sensor networks (WSNs) play an important role in several applications generating a large amount of data. Various applications require continuous updates of critical information—for example, the video streaming of security cameras. These applications need huge spectrum resources. However, the radio spectrum becomes congested by different applications and available channels become scarce. Cognitive radio (CR) has gradually emerged in order to solve the channel scarcity problem [1,2,3].

CR ensures a high spectral efficiency by sharing the frequency band between two kinds of users: primary user (PU) and secondary user (SU). In CR networks, a SU can access, in an opportunistic way, an idle channel licensed to a PU. In order to avoid any harmful interference to the PU, the SU must sense the channel to keep aware of the PU status. When the PU resumes its activities, the SU should immediately vacate the channel. Therefore, the SU should be equipped with a spectrum sensing (SS) unit [4,5,6]. IEEE 802.22 is a CR-based standard used for CR applications. It uses white spaces in the television (VHF/UHF TV broadcast bands) within various frequency bandwidths, such as 54 MHz and 862 MHz, potentially in the 1300 to 1750 MHz and 2700 to 3700 MHz [7].

Applying CR in WSN results in the cognitive-radio-based WSN (CRSN), where the nodes behave as SUs. Thus, they should be aware of their channel status by performing SS to monitor the PU activity. Even the incorporation of CR capability in WSN enhances the spectrum efficiency by exploiting the unused or less used spectrum; this efficiency increase comes at the cost of an additional power consumption due to the performed sensing operations [8,9].

In WSN, clustering, multihop routing (MHR), and data aggregation are efficient techniques adopted to reduce the transmission energy and to maintain the transmission quality and reliability [10,11,12,13,14]. They reduce the transmission radius of the nodes, as each one sends its data via the cluster head (CH) to the central base station (BS). Each node in a CRSN may perform various physical sensing, such as temperature, humidity, moisture, etc., and then transmits its data toward its designated CH. Distances among the nodes and their CHs can be decreased by increasing the number of clusters in the WSN. In addition, clustering decreases the redundancy of the nodes’ data, since the CH may apply an in-network data aggregation (IDA) procedure (based on the mean, the max, or the min of the received data [15]) from the nodes and only transmits aggregated data, using MHR, to the next nearest CH toward the BS, which is the final destination. Like WSN, CRSN nodes consume energy to perform several operations: physical sensing, clustering election, data transmission, etc. In addition to these operations, CRSN consumes energy to perform SS. This inevitable function becomes an energy consuming challenge for the CRSN. Our work focuses on the two potential energy consumption sources, SS and data transmission. Other energy consumption sources are ignored, since the data transmission is the most consuming one in a traditional WSN [16]. In our model, the SS is the exclusive responsibility of CH. Before gathering the data from the cluster nodes and previous CHs, CH should perform SS to avoid any interference with the PU. As mentioned previously, a small number of clusters leads to high energy consumption during the data transmission phase due to the distance among CHs. On the other hand, a big number of clusters leads to drain in energy during the SS phase. Furthermore, the CHs perform IDA in order to reduce the data redundancy. This will impact both the size of the data and the transmission duration. Accordingly, the collision rate between the PU and SU transmissions is influenced by the data aggregation as well as the latency of the data delivery.

In this paper, we investigate the optimization of the CHs number within a CRSN given several parameters, such as the IDA, the energy consumption by physical sensing, energy consumed during the transmission, the SS performance that fulfils the predefined protection level for the PU against the interference of the CRSN nodes, and the high spectrum efficiency to be exploited by these nodes. The existing research works in the literature did not explicitly address the role of SS in determining the optimal number of clusters in a CRSN. The SS requirements should be respected since the CRSN operates as secondary network, and it should respect minimum tolerance of collision with the primary network. Furthermore, to the best of the authors’ knowledge, this is the first research study that addresses the impact of IDA on the collision rate between primary and secondary transmission, the average number of the relayed packets by each CH, and the network latency. The main contributions of this paper are summarized as follows:

1. Seeking an optimal number of clusters of CRSN that takes into consideration several parameters, such as the energy efficiency related to the transmission and the sensing operations, detection, and false alarm probabilities of clustered CRSN.
2. The average number of relayed packets by each CH is obtained in a closed form.
3. Probability of collision and the latency of our energy-optimal clustered CRSN are derived in closed forms, taken into consideration the data redundancy reduction thanks to the IDA.
4. Numerical results are calculated to show the efficiency of choosing the optimal number of clusters, and to figure out the related collision rate and the latency.

The rest of the paper is organized as follows. In Section 2, we present some related works. In Section 3, we present the adopted system model with essential parameters related to the nodes’ distribution, the ad hoc communication, and the data reduction of the transmitted data due to their similarity among neighbor clusters. Problem formulation is detailed in Section 4, where the optimal number of clusters within a CRSN is derived in addition to the optimal value of the consumed energy of the node and the lifespan of the network. In Section 5, mathematical closed forms for both the collision probability and the data delivery latency are given. Numerical results are shown in Section 6 and they corroborate the efficiency of adopting the strategy of selecting the optimal number of clusters, and reveal critical parameters, such as the lifetime of the network, the collision, and the latency. In Section 7, we conclude our work and introduce future perspectives.

## 2.  Related Works

By integrating CR features in WSN, sensor nodes in the CRSN can make the best use of available spectrum resources. However, other challenges have arisen that necessitate investigation in order to identify effective solutions. Clustering, routing, and data aggregation are all significant areas of research and solution development.

Clustering and MHR based networks are addressed in several works in WSN. In [17], the authors proposed a protocol to improve the setting up cluster and the data transmission route. Their simulations showed the effectiveness of the proposed algorithm, which extends the network lifetime and reduces the energy consumption. Thus, the authors of [18] proposed a clustering routing algorithm that prohibits nodes with a low residual energy from becoming CH. In addition, the residual energy is assumed to be an energy control factor to balance the energy consumption.

In [19], the authors propose an adaptive clustering routing algorithm for WSNs to solve the scalability problem in a large-scale case for WSNs. This algorithm divides the network nodes into clusters; then, it selects one node inside each cluster to play the role of CH. Finally, MHR paths are set according to the energy balance principle of the algorithm based on the residual energy, the load, and the number of nodes in the network. In [20], the authors use the Gustafson–Kessel algorithm, which optimizes the number of clusters in order to reduce the energy consumption based on the shape and the volume of clusters, the initial setup of a clustering algorithm, the distribution of the data objects, and the number of clusters. The authors of [21] showed that the optimal number of cluster heads (ONCH) algorithm over LEACH (the low-energy adaptive clustering hierarchy algorithm) is better than LEACH without ONCH as the energy consumption is reduced and the life of the sensor network is extended.

Clustering and MHR have also been addressed for CRSN due to their effect on the secondary interference to the PU as well as the energy efficiency. The authors of [22] proposed a novel clustering process for CRSN in which a pair of nodes close to each other and sharing the same channel can be coupled together. One of these two nodes should go into sleep mode while the other one should stay awake during a single communication interval. Then, they exchange their states after each interval. This technique improves the energy efficiency, as only the awake nodes perform the SS and participate in the election of the cluster head. The authors of [23,24] used the optimal cluster number to improve their groupwise spectrum-aware clustering algorithm. In [23], the authors proposed a novel distributed spectrum-aware clustering (DSAC) algorithm in order to make the spectrum-aware clustering more reliable and practical, while in [24], the authors suggested an optimal cluster number to build up their energy-aware cluster-based routing protocol. In these works, the optimal number of clusters was obtained based on the energy consumed in an intracluster communication related to a phase of gathering data in each cluster, and the energy consumed while transmitting data in intercluster from each CH to the BS in a single-hop transmission. Using MHR [11], the number of relays increases with the hops between sources and destinations [25].

In our work, CH plays the role of a relaying node. Therefore, in order to reduce the transmission range, we must increase the number of clusters and CHs in the network. On the one hand, the energy consumption decreases in MHR-based networks because the radio transmission distance decreases. The collision with the PU may be reduced due to restricted transmission range. On the other hand, the latency and the error probability may increase [26]. However, another factor should be taken into consideration in CRSN, which is the SS. Thus, the optimal number of clusters in a CRSN should be obtained by optimizing the energy consumption, but also, by maintaining a target performance of the SS, the energy consumption in our system is compared to the energy consumption when using the optimal number of cluster calculated in DSAC.

On the other side, data aggregation is one of the most important compression techniques used for wireless networks. This technique reduces the energy consumption during the data gathering phase and accelerates it due to parallel data collection (each CH collects data independently and simultaneously with other CHs in the network). Data aggregation for CRSN was addressed in several works such [27,28,29,30]. The authors of [27] incorporated the basic CRSN characteristics in an information theoretical capacity maximization framework with the combination of energy-adaptive mechanisms and the information correlation for a multihop CRSN topology. A small network is used in their simulations (three sensors by hop and three hops toward the sink); it was concluded that an energy-adaptive mechanism outperforms a nonadaptive mechanism. They also concluded that the networks that use information correlation in data aggregations outperform the other networks. Works of [28,29,30] addressed the minimum latency data aggregation scheduling (MLDAS). In [28], a dense CRSN was considered. The authors assumed an unsymmetrical communication links among SUs and a possible SU–SU collision. To reduce the number of collisions and better sharing of the spectrum resources, SU should be in a competitor mode. Two practical distributed algorithms under a unit disk graph interference model and physical interference model were proposed in order to investigate the MLDAS problem. The authors of [29] focused on a probabilistic network model, i.e., links are not always guaranteed between nodes. Two networks are considered: a regular wireless network (RWN) with regular users and an auxiliary wireless network (AWN) with auxiliary users. Any user may send its data over the RWN spectrum with an equal chance with other users, or it can opportunistically operate on AWN if and only if the AWN spectrum is not occupied by any auxiliary user. The authors of [30] constructed the aggregation tree and the computation of a conflict-free schedule simultaneously without any predetermined structure. MLDAS was extended to multiple-channels access. Additionally, a scheduled node cannot participate in the aggregation process; thus, a new aggregation mode called data aggregation scheduling in the dark was proposed in order to utilize the spectrum opportunities of scheduled nodes.

As previously stated, numerous researchers have proposed alternative approaches to tackle major challenges facing the CRSN system. One can also notice the tremendous efforts and the persistent pursuit to reduce energy consumption and maximize the lifespan of the CRSN. In this paper, we introduce the energy consumed during the SS procedure into an optimization problem in order to find the best number of clusters that extends the network lifespan. As we will see later, SS is a costly operation that, if not properly considered, might result in an early energy depletion.

## 3. CRSN’s System Model

Let us consider a CRSN operating opportunistically over a licensed channel reserved to a PU. For the sake of simplicity, we assume a deterministic network model, where the node links are stable, and symmetrical communication links between SUs are considered. The CRSN contains *N* homogeneous sensor nodes [31] distributed over a large circular geographical zone [32] with a radius *R*. In this zone, all physical records should be transmitted toward a BS located at the center [33]. The CRSN is assumed operating on a band allocated to a PU, i.e., the PU activity throughout all the CRSN is homogeneous. Such an assumption is suitable to the realistic scenarios means that the PU coverage is large and the whole CRSN falls into this coverage, e.g., the Global System Mobile (GSM) network [34]. Another case study is the radio FM or TV white space, where the same frequency may be used by the same PU within a whole country [35].

**Figure 1:**
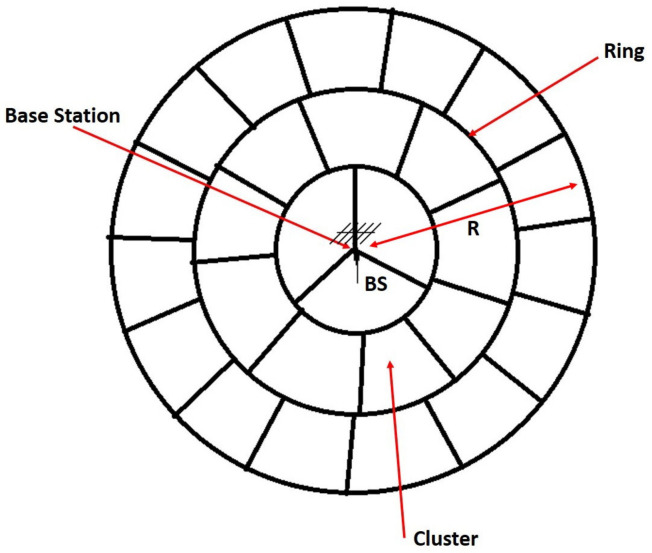
Clustering network with equal cluster size where k=3 is the number of rings splitting a network of radius *R*.

As shown in Figure 1, the network is divided into a set of *k* rings, called SS rings in reference to spectrum sensing. Rings are spaced by $\frac{R}{k}$, and each ring is divided into several clusters called SS clusters with equal size. We suppose that the first ring (near the BS) contains three clusters; then, the area of each cluster is equal to: $\frac{\pi }{3}{\left(\frac{R}{k}\right)}^{2}$. The area of the *i*th ring can be evaluated as follows:

(1) $$\begin{array}{cc} A_{i} & =\pi {\left(i\frac{R}{k}\right)}^{2}-\pi {\left(\left(i-1\right)\frac{R}{k}\right)}^{2}\\ \; & =\pi \left(2i-1\right){\left(\frac{R}{k}\right)}^{2} \end{array}$$

where i∈[1,k] is the index of the ring. Hence, the *i*th ring contains a total number $m_{i}$ of clusters equals to:

(2) $$m_{i}=\frac{\pi {\left(\frac{R}{k}\right)}^{2}\left(2i-1\right)}{\frac{\pi }{3}{\left(\frac{R}{k}\right)}^{2}}=3(2i-1)$$

Notice that the closest ring to BS (i=1) contains three clusters.

Using Equation (2), we can find the total number of clusters *M* in the overall network:

(3) $$M=\sum_{i=1}^{k}3\left(2i-1\right)=3k^{2}$$

**Figure 2:**
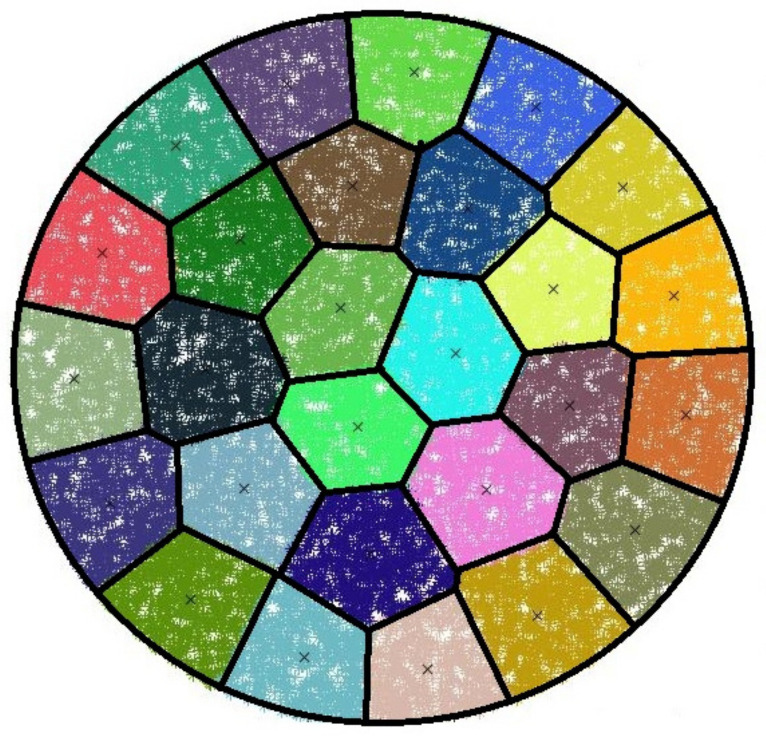
*K*-means applied on a circular CRSN uniformly distributed; the number of cluster was predefined.

At this point, the clustering algorithm with all its features (clustering mechanism, clustering cycle, and wake-up scheme) is beyond the scope of our investigation. In our research, we assume that clusters are initially existing and evenly distributed over the whole network. The topology of our adopted system model is inspired from the behavior of the well known *K*-means clustering algorithm when applied to a circular WSN. Figure 2 gives an example of *K*-mean clustering, in which the topology may be approximated to our model with three rings. As shown in this figure, the ring around the origin (where the BS is located) contains three clusters, similarly to our system model.

Our network is considered as a homogeneous network. However, we can also extend our work on a heterogeneous network with *A* nodes where only *N* nodes, N≤A, can be elected as CHs, because they have the required capability to fulfill the CH role.

In our model, a multihop transmission system is adopted among the CHs. Thus, the message sent from a far CH to BS should pass by the CHs in between.

**Figure 3:**
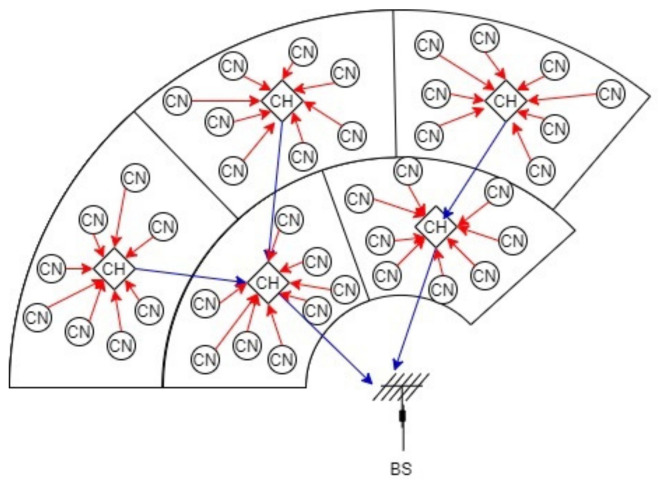
Gathering data from CNs and CHs.

According to our model, CRSN carries out an IDA technique [36], where CH gathers the data of the nodes inside its cluster and aggregates the data collected from farther CHs. Figure 3 shows an example of the process of collecting data. Nodes, other than CHs, are called cluster nodes (CNs).

CNs collect data on a regular basis and send it to CHs whenever requested. In turn, CH makes SS, if the channel is free; then, it requests data from their CNs as well as from neighboring CHs. All CNs transmit their packets toward their appropriate CH. In order to avoid collision among CHs transmission, CSMA/CA and TDMA techniques can be used [37].

**Figure 4:**
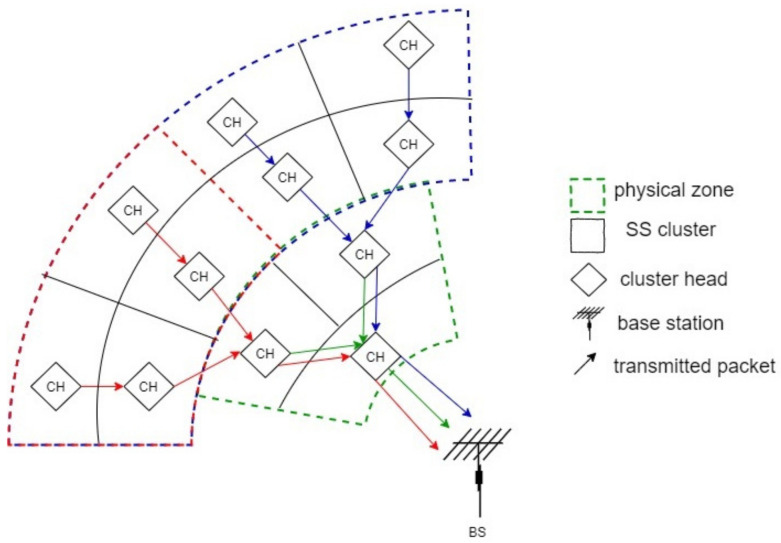
Data routing between CHs located in different similar physical value zones.

Generally, CNs and CHs close to each other may collect similar physical values, such as humidity, temperature, etc. Figure 4 shows a part of CRSN divided into clusters and virtual physical zones with similar clusters (dashed lines) that have similar data packets. The network is also divided into virtual physical rings, and contains $k_{p}$ physical rings. Each physical zones contains $\frac{k}{k_{p}}$ rings where $k_{p}\leq k$. In average, each physical ring contains a number of physical zones, formed by an equal number of SS clusters. Let our CRSN has *P* similar physical value zones generating in average *P* distinct packets in the entire CRSN. Each physical zone contains several SS clusters. Hence, the maximum number of distinct packets retransmitted by the nearest CHs to the BS is $\frac{P}{3}$, where 3 is the number of clusters corresponding to the first ring (see Figure 1). In our system, a cluster receives the packets coming from its previous CHs’ peers. Then, it compares received packets with each other and with its own packet collected from its cluster members. Subsequently, it compacts the similar packets into a single packet by counting the number of the occurrence and merging them into a new packet before sending it. In the end result, this cluster sends packets that differ from each other by a predetermined threshold amount. There are many methods for calculating the difference between data packets; the method used in our previous work based on the Euclidean distance can be used such as [38]. Figure 4 shows that if a CH receives *p* data packets from a neighbor CH located in the same physical zone, then it retransmits *p* packets to the next CH, whereas if a CH receives *p* data packets from a CH in a different physical zone, it then retransmits p+1 data packets to the forward CH [39].

To detail the IDA process above, after collecting data coming from its CNs and neighboring CHs, a CH performs three steps to reduce the data redundancy, as described in our previous study [38]:

1. CH aggregates the data received from CNs in a fixed size packet [38].
2. The CH applies a similarity test performing IDA process, based on the Euclidean’s norm, applied to all packets.
3. Finally, a CH just transmits distinct packets toward the next CH.

Obviously, CHs are prone to depletion of energy first. Therefore, the CH role is periodically rotated among potential nodes [12,32]. The number of hops from the CH to the BS depends on the distance between them. The ring number in the CRSN can be considered as the maximum number of hops between the farthest CH and BS. A high number of hops means that a low amount of energy will be consumed by the transmission, but the energy consumed by SS will be increased, and vice versa.

Hereinafter, we discuss how to find the optimal number of clusters that saves energy and improves the network lifespan while satisfying an acceptable level of protection for the PU against the interference characterized by a high probability of PU signal detection and achieving high spectrum efficiency by reducing the probability of false alarm on the PU presence.

## 4. Problem Formulation

In WSN, each node collects data from its environment and transmits them toward CH. In our model, a CH has two major roles:

1. Performing the SS before requesting the data from the CNs and its previous CHs.
2. IDA based data gathering and retransmitting from its CNs and its backward CHs as per ad hoc topology.

Therefore, the main energy consumption of the CH comes from two operations:

- When performing the SS operation, an energy amount $e_{s}$ is needed.
- The transmission of the data from a given CH toward the next CH in the direction of destination also consumes energy.

The energy of data transmission depends on the decision outcome of SS performed by CH giving the permission to transmit the data:

1. CH makes a miss detection decision on the PU status, i.e., PU is detected absent while it is truly active. Thus, a collision may take place.
2. CH makes a true decision on the absence of PU. In this case, CH can send its data toward the next CH freely on the PU channel.

Accordingly, the probability that a CH transmits the data is given by:

(4) $$p_{t}=p\left(H_{0}\right)\left(1-p_{fa}\right)+p\left(H_{1}\right)\left(1-p_{d}\right)$$

where $p(H_{0})$ and $p(H_{1})$ are the prior probabilities that the channel is idle or active, respectively. $p_{fa}$ and $p_{d}$ are the desired false alarm and detection probabilities, respectively, ensuring the SS requirements.

The number of SS operations and the transmission range are directly related to the number of rings *k*. Therefore, finding the optimal number of rings *k*, which extends the network lifespan *L*, becomes essential for the network. Furthermore, network lifespan is directly affected by the energy consumption along CHs and CNs in our network. Because of the obvious difference in power consumption between the CHs and the CNs, our problem can be formulated as finding the optimal *k* according to energy consumed by the CHs only.

Let us define $e_{i}$, the average energy consumed by a network node (CH or cluster member) located in the *i*th ring:

(5) $$e_{i}=\left[e_{r}{\left(\frac{R}{k}\right)}^{2}p_{t}N_{p_{i}}+e_{s}+e_{0}N_{p_{i}}\right]\frac{M}{N}+\left[e_{r}{\left(\frac{R}{k}\right)}^{2}p_{t}+e_{0}\right]\left(1-\frac{M}{N}\right)$$

where $N_{p_{i}}$ is the average number of packets relayed by CHs in the *i*th ring, $e_{r}$ is the energy density at the receiver antenna and $e_{r}{\left(\frac{R}{k}\right)}^{2}$ is the amount of energy consumed by CH during the data transmission. *N* is the total number of nodes and $\frac{M}{N}$ denotes the probability of a node to be a CH, and the term multiplied by $\frac{M}{N}$ is the energy consumed by a CH (transmission, SS, running time), whereas $\left(1-\frac{M}{N}\right)$ is the probability of a node to be a CN, and it is multiplied by the energy consumed by the CN (transmitting data to CH, running time). $e_{0}$ is the energy consumed by the node in the running mode.

Let us define $e_{max}$ as the maximum consumed energy per CHs in the CRSN network. In this work, the optimization is performed with respect to the first node dies (FND) criterion. Lifespan is assumed to be the number of iterations that can be performed by the CRSN before the extinction of the first CH in the network [40].

Assuming that each node is initially provided with an amount of energy called the initial energy $e_{int}$. Hence, lifespan *L* can be calculated as follows:

(6) $$\begin{array}{cc} L & =\frac{e_{int}}{e_{max}} \end{array}$$

where:

(7) $$\begin{array}{c} \;e_{max}=\max_{1\leq i\leq k}e_{i} \end{array}$$

The optimization problem, looking for extending the network lifespan, can be defined as follows:

(8) $$\begin{array}{cc} \max_{k\neq 0} & \;\frac{e_{int}}{e_{max}}\\ s.t.\; & M\leq N \end{array}$$

Since $e_{int}$ is constant, the maximization of (8) can be reformulated as a minimization problem as follows:

(9) $$\begin{array}{cc} \min_{k\neq 0} & \;e_{max}\\ s.t.\; & M\leq N \end{array}$$

Using Equation (5), we substitute $e_{max}$ by its value in Equation (9) to obtain:

(10) $$\begin{array}{cc} \min_{k\neq 0}[\max_{i}\left(e_{r}{\left(\frac{R}{k}\right)}^{2}p_{t}N_{p_{i}}+e_{s}+e_{0}N_{p_{i}}\right) & \frac{M}{N}+\left(e_{r}{\left(\frac{R}{k}\right)}^{2}p_{t}+e_{0}\right)\left(1-\frac{M}{N}\right)]\\ s.t. & M\leq N \end{array}$$

It becomes obvious that the closest CHs to the BS (ring 1) consume the largest amount of energy due to the relaying role. Hence, our problem can be reformulated as:

(11) $$\begin{array}{cc} \min_{k\neq 0}[\left(e_{r}{\left(\frac{R}{k}\right)}^{2}p_{t}k_{p}^{2}+e_{s}+e_{0}k_{p}^{2}\right) & \frac{M}{N}+\left(e_{r}{\left(\frac{R}{k}\right)}^{2}p_{t}+e_{0}\right)\left(1-\frac{M}{N}\right)]\\ s.t. & M\leq N \end{array}$$

**Proof.**  See Appendix C. □

Now, replacing *M*, the number of CHs in the network, with its value $M=3k^{2}$ as seen in Section 3 in our problem:

(12) $$\begin{array}{cc} \min_{k\neq 0}[\left(e_{r}{\left(\frac{R}{k}\right)}^{2}p_{t}k_{p}^{2}+e_{s}+e_{0}k_{p}^{2}\right) & \frac{3k^{2}}{N}+\left(e_{r}{\left(\frac{R}{k}\right)}^{2}p_{t}+e_{0}\right)\left(1-\frac{3k^{2}}{N}\right)]\\ s.t. & M\leq N \end{array}$$

**Theorem** **1.** *If f(x) is a unimodal function defined on $G\subset R^{+}$ having a minimum as extremum at $x_{0}>0$, then f(n), n∈{N∩G}, is a set of points having a minimum $M=min\{f\left(\left\lfloor x_{0}\right\rfloor \right),\;f\left(\left\lceil x_{0}\right\rceil \right)\}$.*

where ⌊·⌋ and ⌈·⌉ stands for the floor and ceiling operator, respectively.

**Proof.**  Let f(x) be a unimodal function defined on $G\subset R^{+}$ with $\frac{df}{dx}{|}_{x=x_{0}}=0\;\mathrm{and}\;f\left(x_{0}\right)=\min\left\{f\left(x\right)\right\}\;\mathrm{for}\;x\in G$. Let n∈{N∩G} be a natural variable and $U_{n}=\left\{f\left(n\right)\;|\;n\leq x_{0}\right\}$ and $V_{n}=\left\{f\left(n\right)\;|\;n\geq x_{0}\right\}$ are two numerical sequences, knowing that $U_{n}$ is monotonically descendant, and $V_{n}$ is monotonically ascendant; thus, $f(\left\lfloor x_{0}\right\rfloor )$ and $f(\left\lceil x_{0}\right\rceil )$ are the minimum of $U_{n}$ and $V_{n}$ at $\left\lfloor x_{0}\right\rfloor$ and $\left\lceil x_{0}\right\rceil$, respectively. Obviously, the min of f(n) is $M=\min\{\min\left\{U_{n}\right\},\min\left\{V_{n}\right\}\}$; hence, $M=\min\{f\left(\left\lfloor x_{0}\right\rfloor \right),\;f\left(\left\lceil x_{0}\right\rceil \right)\}$. □

Using Theorem 1, we can show that our Equation (12) can be associated to a unimodal function with a single minimum on its defined domain. Thus, our problem can be simplified into a simple optimization problem. Replacing *k* with a continuous variable *x* in Equation (12), and differentiating $e_{i}$ with respect to *x*, we obtain the optimal value:

(13) $$\begin{array}{c} k_{opt}=argmin\left(e_{\left\lfloor R\sqrt[{4}]{\frac{e_{r}p_{t}D}{e_{s}+e_{0}\left(k_{p}^{2}-1\right)}}\right\rfloor },e_{\left\lceil R\sqrt[{4}]{\frac{e_{r}p_{t}D}{e_{s}+e_{0}\left(k_{p}^{2}-1\right)}}\right\rceil }\right) \end{array}$$

where ⌈.⌉ stands for the ceiling operator and ⌊.⌋ stands for the floor operator. if $k_{opt}>>1$, we can approximate $k_{opt}$ to:

(14) $$\begin{array}{c} k_{opt}=\left\lfloor R\sqrt[{4}]{\frac{e_{r}p_{t}D}{e_{s}+e_{0}\left(k_{p}^{2}-1\right)}}\right\rfloor \end{array}$$

where $D=\frac{N}{\pi R^{2}}$ is the density of the network. Since FND is adopted in this work, the optimal value $e_{opt}$ of $e_{max}$ is given by substituting $k_{opt}$ in Equation (5):

(15) $$e_{opt}=2\sqrt{\frac{e_{r}p_{t}e_{s}}{D}}+\left(k_{p}^{2}-1\right)\left(e_{0}\sqrt{\frac{e_{r}p_{t}}{e_{s}D}}+\frac{e_{r}p_{t}}{D}\right)$$

**Figure 5:**
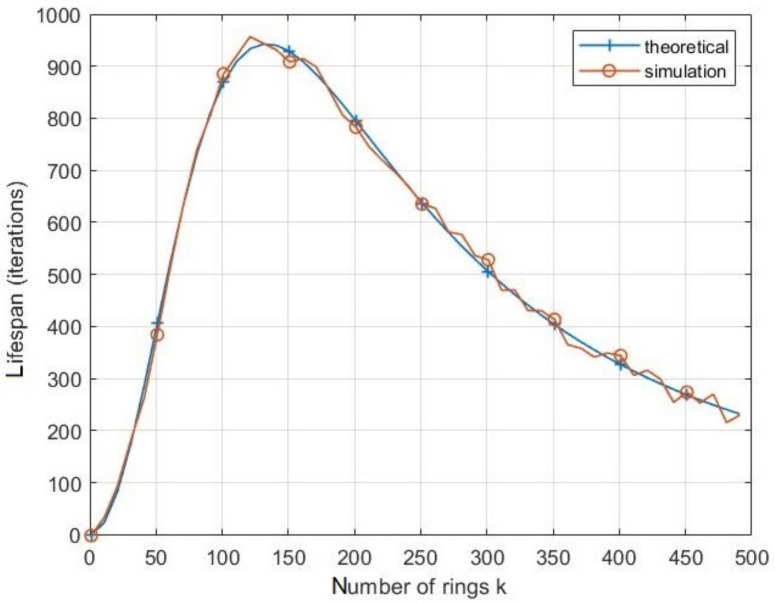
Theoretical and simulation results of the lifespan vs. number of rings in CRSN.

Figure 5 shows the lifetime of the nearest node with respect to the number of clusters. As it can be seen, the theoretical results coincide with the simulated ones approving Equation (5). Lifespan increases until reaching a maximum before decreasing again. Indeed, when the number of clusters is low, the size of a cluster becomes large, then nodes consume a great amount of transmission energy. Subsequently, the energy consumed while transmission becomes greater than the energy consumed to perform the SS by CH. As the number of clusters increases, their size decreases; hence, the transmission energy consumed by CHs decreases. Therefore, the lifespan increases until a maximum value at a specific value of *k* is reached. However, if the number of cluster continues to decrease, the energy consumed by SS is considerably high compared to the low transmission energy, so the lifespan decreases.

The number of clusters is related directly to the number of rings *k* as shown in Equation (3). Hence, the optimal value $k_{opt}$ of *k* should maximize the lifespan of the network.

## 5. Network Latency and Collision Probability

In this section, we derive the average time taken by each round of data gathering in the network, and the probability of collision with the PU. Furthermore, using the analytical expressions of the collision and the latency, we prove analytically that the first ring (k=1) of the network spends the larger amount of energy across the network.

### 5.1. Collision Probability and Latency

Each time the BS requests data from nodes, the CNs start sending to the CHs toward the BS. In turn, before receiving data from CNs and backward CHs, CH should sense the channel in order to ensure that PU is absent to avoid any collision.

**Figure 6:**
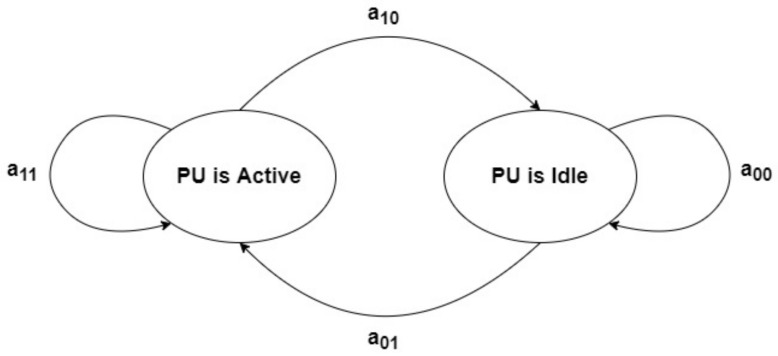
The PU activity modeled as a two-state Markov chain.

The state of PU (active or idle) is modeled using a discrete Markov chain as shown in Figure 6 [41], where $a_{ij}$ is the transition probability of the PU from the state *i* to the state *j*. The time in this Markov chain is divided into equal slots of $\tau_{s}$ seconds.

Each CH, acting as SU, has four tasks to do:

1. Performing SS to avoid any collision with PU.
2. Broadcasting a message to CNs and neighboring CHs indicating that it can receive collected data.
3. Gathering data from CNs and neighboring CHs and aggregating that data.
4. Waiting a broadcasting message from the next CH before transmitting packets toward it.

The data sending starts from the CHs of the farthest ring to the next one toward the BS. The probability that a collision occurs during the data transmission from the farthest ring until the BS is given by:

(16) $$P_{c}=1-\prod_{i=1}^{k}\left(1-P_{c_{i}}\right)$$

where $P_{c_{i}}$ is the probability of collision with PU in the *i*th ring in a network. Such a collision may take place in one of the following scenarios:

1. A miss detection decision on the PU presence is made by the CH.
2. The CH makes a correct decision on the PU absence, but the latter resumes its activity during the transmission period of the CH.

Consequently, $P_{c_{i}}$ could be evaluated as follows:

(17) $$P_{c_{i}}=P\left(H_{1}\right)\left(1-p_{d}^{m_{i}}\right)+P\left(H_{0}\right)\left(1-p_{fa}^{m_{i}}\right)\left(1-a_{00}^{\frac{T_{a_{i}}}{\tau_{s}}}\right)$$

$a_{00}$ is the transition probability that the PU will stay in the idle state at the next time slot $\tau_{s}$, and $T_{a_{i}}$ is the average time of transmission activity of CH in the *i*th ring:

(18) $$\;T_{a_{i}}=\frac{N\times T_{CN}}{3k^{2}}+\tau_{T_{i}}V_{i}$$

where *N* is the total number of CNs in the overall CRSN, $T_{CN}$ is the period of transmission data packet from a single CN to its appropriate CH, and $\tau_{T_{i}}$ is the average time of collecting data of a CH in ring *i* from one of its $V_{i}$ previous CHs in the previous SS ring i+1. Equation (18) reflects the adoption of the time division multiple access (TDMA) technique by the CNs, as only one channel is assumed to be used by these nodes to send their data to CH. TDMA, in our case, prevents the interference between the transmissions of the CNs and lets the CH collect the data properly.

As mentioned previously, $V_{i}$ is the average number of CHs that transmit data packets to a single CH in the ring *i*, in other words, the average number of previous CHs of any CH in the *i*th ring in our CRSN. This value can be calculated as follows:

(19) $$V_{i}=\frac{3(2(i+1)-1)}{3(2i-1)}=\frac{2i+1}{2i-1}$$

where i∈[1,k−1].

Neighbor CNs may collect similar data. Therefore, we assume that there are *P* distinct physical zones with similar areas. Thus, the average time of transmission $\tau_{T_{i}}$ of each CH member of ring *i* can be presented as follows:

(20) $$\tau_{T_{i}}=\frac{T_{P}}{m_{i}}\sum_{j=\left\lceil \frac{k_{p}}{k}i\right\rceil }^{k_{p}}\sum_{t=1}^{m_{i}}\left(\left\lceil \frac{mp_{j}}{m_{i}}t\right\rceil -\left\lfloor \frac{mp_{j}}{m_{i}}\left(t-1\right)\right\rfloor \right)$$

where $T_{P}$ is the period of a single CH packet transmission, $k_{p}$ is the number of physical ring, $m_{p_{j}}$ is the number of physical zones in a physical ring *j*, and $m_{i}$ is the number of SS clusters in the *i*th ring.

**Proof.**  See Appendix A. □

Equation (20) can be rewritten as:

(21) $$\tau_{T_{i}}=\frac{T_{P}}{m_{i}}\sum_{j=\left\lceil \frac{k_{p}}{k}i\right\rceil }^{k_{p}}\left(m_{p_{j}}+m_{i}-m_{p_{j}}\wedge m_{i}\right)$$

with:

(22) $$m_{p_{j}}+m_{i}-m_{p_{j}}\wedge m_{i}=\sum_{t=1}^{m_{i}}\left(\left\lceil \frac{mp_{j}}{m_{i}}t\right\rceil -\left\lfloor \frac{mp_{j}}{m_{i}}\left(t-1\right)\right\rfloor \right)$$

where (m∧n) stands for the greatest common divisor operator of *m* and *n*.

**Proof.**  See Appendix B. □

Consequently, the period of overall collecting data cycle from the network becomes:

(23) $$\begin{array}{cc} T_{c} & =\frac{1}{P\left(H_{0}\right)\left(1-p_{fa}\right)}\sum_{i=1}^{k}T_{a_{i}}\\ \; & =\frac{1}{P\left(H_{0}\right)\left(1-p_{fa}\right)}\sum_{i=1}^{k}\left[\tau_{B}+\frac{NT_{CN}}{3k^{2}}+\frac{T_{p}V_{i}}{m_{i}}\sum_{j=\left\lceil \frac{k_{p}}{k}i\right\rceil }^{k_{p}}m_{p_{j}}+m_{i}-\left(m_{p_{j}}\wedge m_{i}\right)\right] \end{array}$$

In Equation (23), we assume that any transmission between two CHs, or CNs and CHs, cannot succeed in presence of PU; Therefore, we divide the term $\sum_{i=1}^{k}T_{a_{i}}$ (the total time of collecting data without interruption of PU) over the probability of transmission data without presence of PU.

### 5.2. Maximal Consumed Energy

Regarding Equation (15) on $e_{opt}$, in order to prove that the CHs in the first ring consume the maximum amount of energy among all other CHs, we can instead prove that the CHs in that ring relay the maximum number of packets toward the BS. The average number of packets relaying by CHs in the *i*th ring, $N_{p_{i}}$ can be derived from Equation (21):

(24) $$N_{p_{i}}=\frac{1}{m_{i}}\sum_{j=\left\lceil \frac{k_{p}}{k}i\right\rceil }^{k_{p}}\left(m_{p_{j}}+m_{i}-m_{p_{j}}\wedge m_{i}\right)$$

Therefore, each CH in the ring *i* relay $N_{p_{i}}$ packets, in this case, a CH at the first ring should aggregate $N_{p_{1}}$ packets:

(25) $$N_{p_{1}}=\frac{1}{3}\left(\sum_{j=1}^{k_{p}}3\left(2j-1\right)+3\sum_{j=1}^{k_{p}}1-\sum_{j=1}^{k_{p}}\left(3\left(2j-1\right)\wedge 3\right)\right)=k_{p}^{2}$$

Hereinafter, we show that:

(26) $$N_{p_{1}}>N_{p_{i}},\;\forall i>1.$$

**Proof.**  See Appendix C. □

## 6. Numerical Results

In this section, we investigate our theoretical results and show the effectiveness of the IDA in reducing both the collision probability and the network latency. The results are compared with the classical CR without IDA techniques, and we compare our energy consumption, data latency, and collision probability with the DSAC system [23].

In our simulations, we consider a circular network containing *N* nodes with a radius R=100 m and 3 physical similar rings. According to Equation (A2) in Appendix A, the number of physical zones is P=27. The processing energy of nodes during the receiving is $e_{0}=50$ nJ/packet and the transmission energy constant is $e_{r}=0.1$ nJ/bit/m^2^ [42]. For the sensing process, a SNR of −1 dB is considered, and $p_{d}=0.9$ and $p_{fa}=0.1$ are set as the target probabilities to meet the SS requirement. An energy detection method is considered for SS, and the consumed energy by the sensing $e_{s}$ is evaluated according to the number of samples $N_{s}$ needed for the sensing process [43] and the energy consumed per sample $E_{s}$:

(27) $$e_{s}=N_{s}E_{s}$$

**Figure 7:**
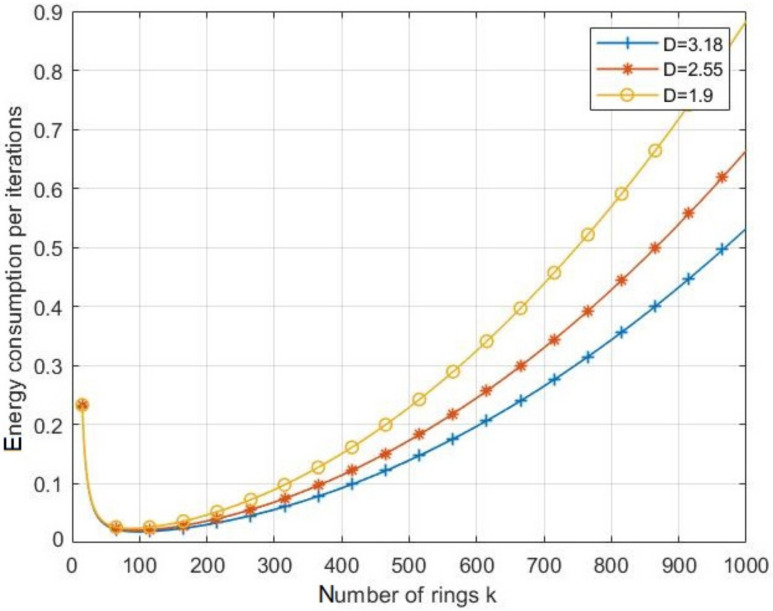
The effects of the ring number on the energy consumption per iteration on $e_{max}$ with different network densities.

Figure 7 depicts the effects of the ring number *k* on the energy consumption by iteration with different values of node density *D*. It can be shown that increasing the density of nodes inside the network leads to decrease the energy consumed during an iteration. As for the impact of the number of rings *k*, the energy consumption per iteration decreases with the increase of *k* until the latter reaches an optimal value, at which point this energy consumption becomes minimal. If *k* continues increasing, the energy consumption per iteration reincreases, leading to shortening of the network lifespan.

**Figure 8:**
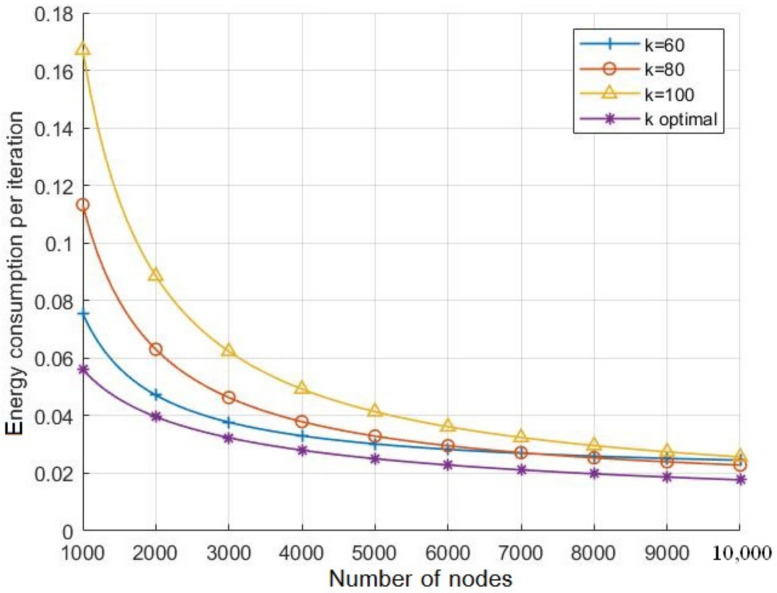
The effects of the number of nodes on the energy consumption with different values of *k*.

**Figure 9:**
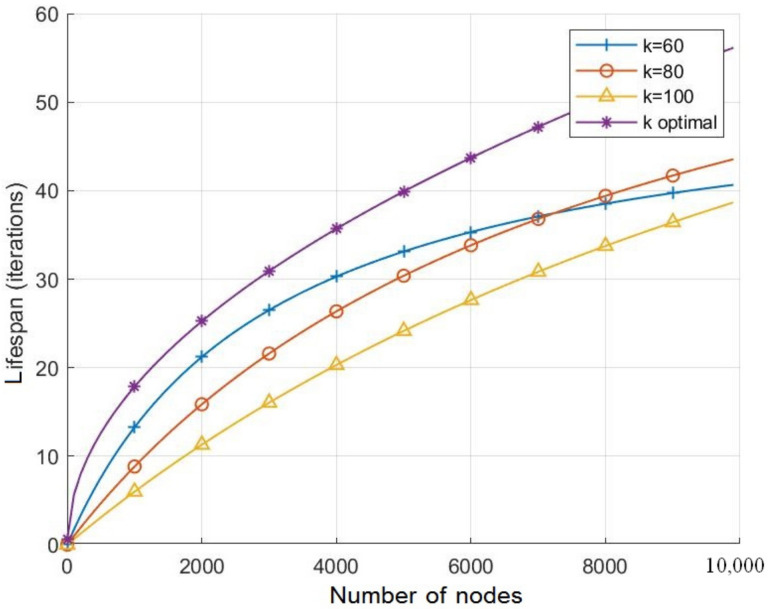
The effects of the number of nodes on lifespan with respect to various values of *k*.

**Figure 10:**
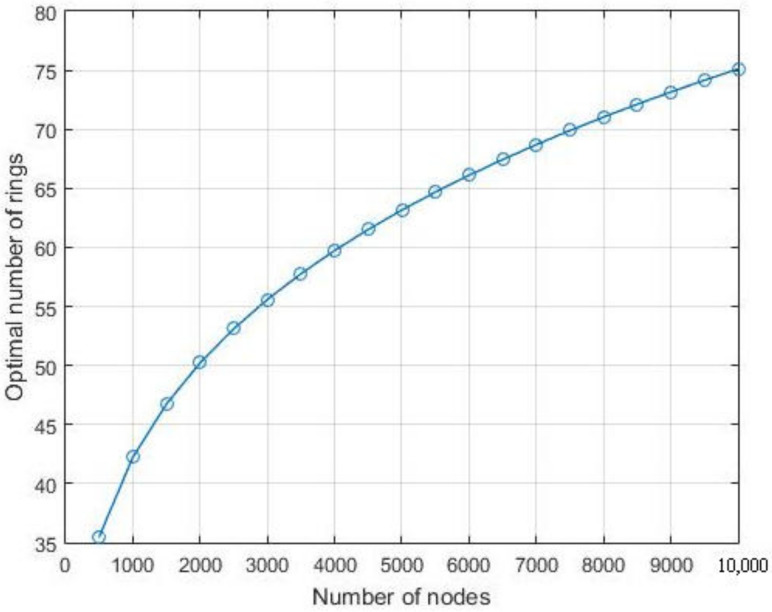
The optimal number of rings *k* vs. the node numbers.

Figure 8 and Figure 9 highlight the effects of the nodes number on the energy consumption and lifespan respectively for various values of *k* including the optimal value. As the two figures show, for all the considered values of *k*, the energy consumption per iteration decreases with respect to the number of nodes while the network lifespan increases. However, when setting *k* to its optimal value for each nodes number *N*, Figure 8 shows the energy consumption per iteration decreases compared to the other considered values of *k*, which leads to extend the network lifespan as presented in Figure 9. Note that, with a node number less than 7000, the lifespan with k=60 is longer than the one observed with k=80, but it appears shorter when the number of nodes is more than 7000. In fact, with less than 7000 nodes, the optimal number of *k* becomes closer to 60. However, this number increases with the number of nodes, and it approaches to 80 when the number of node is above 7000, as shown in Figure 10.

Hereinafter, we investigate the performance of the CRSN with and without IDA in terms of the collision probability and the data delivery latency. The numerical results are based on the data size effect. Indeed, other message contents, such as the header and the trailer of the message, may affect the performance, but these contents are not considered in our work.

**Figure 11:**
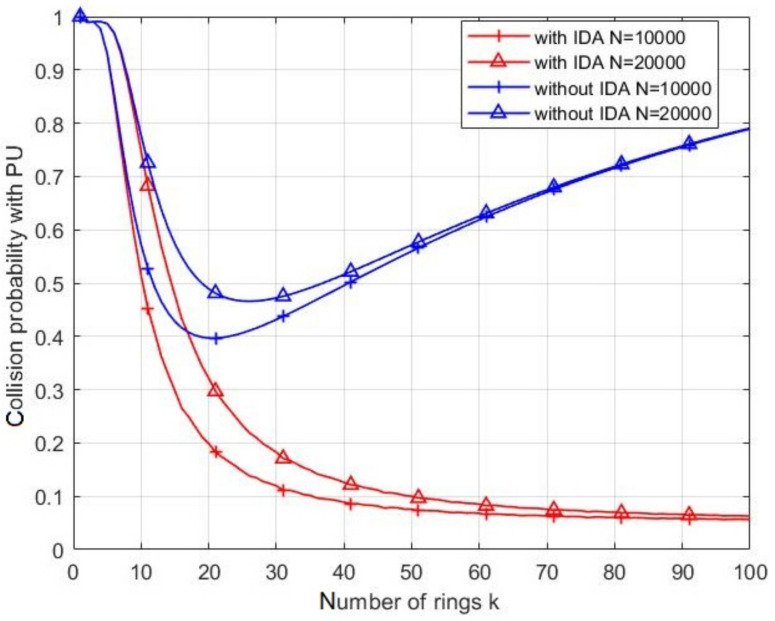
The collision probability of CH with PU during its activity with and without IDA.

The collision probability of CHs with PU in the middle ring (ring $\left\lceil \frac{k}{2}\right\rceil$) of the network during the PU activity, with and without IDA, are shown in Figure 11. For both cases (with and without IDA), the collision rate increases with *N* due to the increasing number of messages to be transmitted. When IDA is applied, the probability of collision with PU decreases with respect to the number of rings. This effect is due to both the aggregation of the messages and the reduction of the number of CNs in each cluster. Hence, the time of collecting data in each cluster decreases leading to decrease the probability of collision. By contrast, without IDA, the probability of collision increases after reaching a minimum value. This refers to the huge number of packets retransmitted by each CH coming from the previous CHs. Without IDA, the packet length increases linearly with the number of rings and results in long transmission time of CHs.

**Figure 12:**
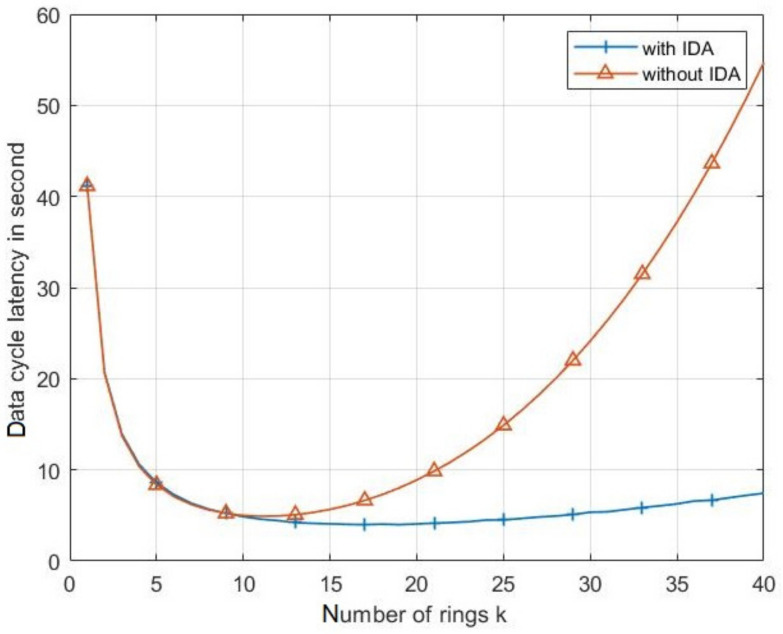
The duration of the complete data collection cycle from CN to the BS with and without IDA.

Figure 12 shows the duration of the complete collecting data cycle from CN to the BS. As clearly shown in this figure, IDA leads the CRSN to highly reduce the data collection cycle period compared to the case without IDA. For all cases and for a small number of rings, the CH spends a great amount of time collecting data from its cluster nodes. This time decreases with an increasing *k* until the latter surpasses a certain value. Beyond this value of *k*, collecting time increases again. This effect is due to the multihop transmission, where a big number of routes results from the high values of *k* and procures a big time of transmission between CHs and BS.

**Figure 13:**
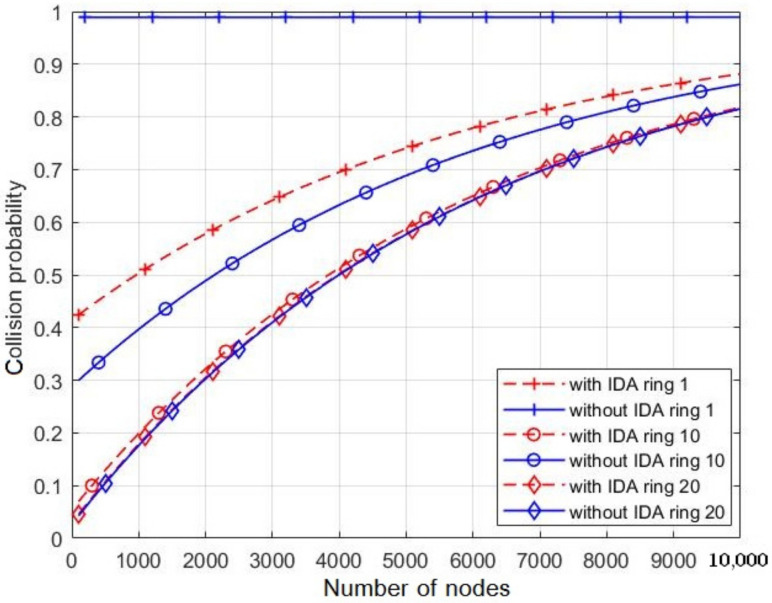
The collision probability of CH with PU during its activity with and without IDA with different rings.

Figure 13 depicts the collision probability with and without IDA, during a transition of rings: k=1, k=10, and k=20, with respect to the number of nodes in the network. Clear effect of the IDA can be noticed on reducing the collision compared to case of no IDA for the three considered values of *k*. For instance, for k=1 and N=1000, the collision rate is about 0.5 when IDA is adopted, whereas it is closed to 1 for no IDA. However, the collision rate exhibits an increasing with *N* for both cases due to the increase in the number of messages to send.

**Figure 14:**
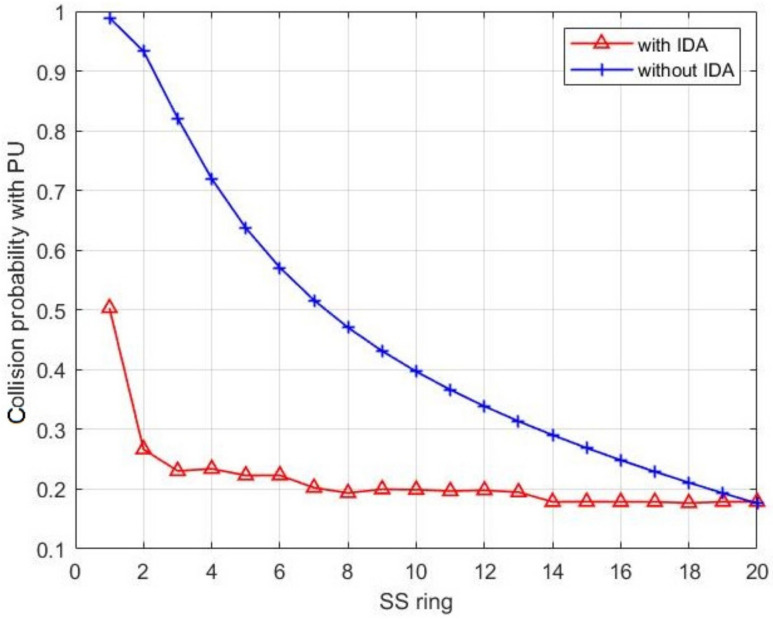
The collision probability of CH with PU during its activity among all network rings.

Figure 14 shows the collision probability in networks, with and without IDA, in different rings where the number of nodes is fixed to N=1000. It can be shown that, at the nearest ring from the BS (ring 1), we could notice a big difference between the two curves due to the redundancy reduction of the message obtained when IDA is applied. This must reduce the message length and, thus, alleviate the collision rate. Moving forward to the farthest ring from the BS (in our example it is ring 20), the curves begin to converge until they intersect at the last ring. This is because the collision with PU at the last ring during the SU activity is the same for both cases, with and without IDA, since there are no packets coming to the last ring, i.e., the CHs at this ring do not relay any packet from previous rings.

**Figure 15:**
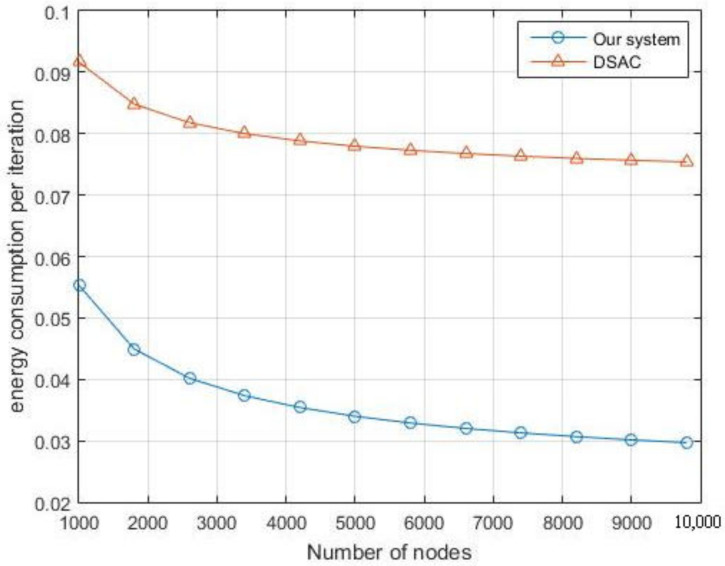
Comparison of energy consumption between our system and the DSAC.

In Figure 15, we compare our system with the DSAC system mentioned in [23]. The DSAC authors have calculated the optimum value for the number of clusters in the network based on the maximum transmission distance at the sensor nodes and the density of the sensor nodes in the network. We should highlight that our simulations showed that the energy consumption in our system is significantly lower than that of DSAC. The reason here is because we have taken into consideration the amount of energy consumed during the SS process when we optimize our problem.

**Figure 16:**
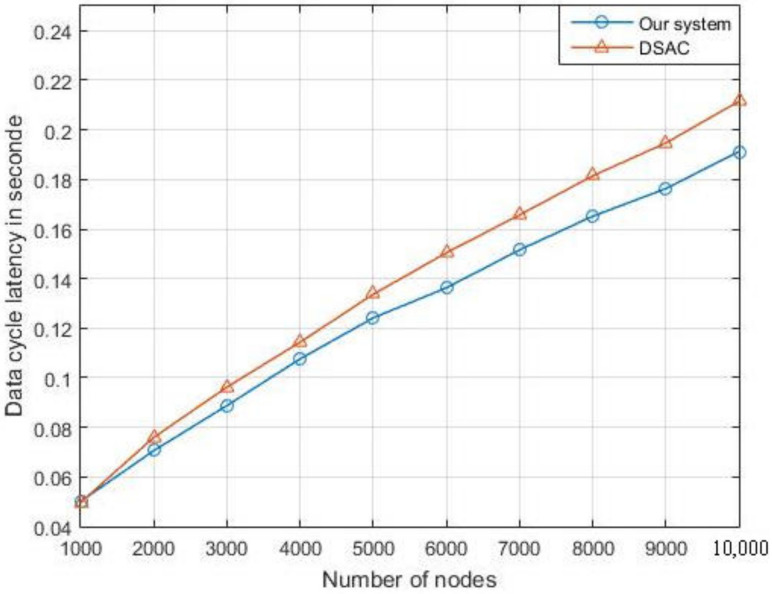
Comparison of data latency between our system and the DSAC.

**Figure 17:**
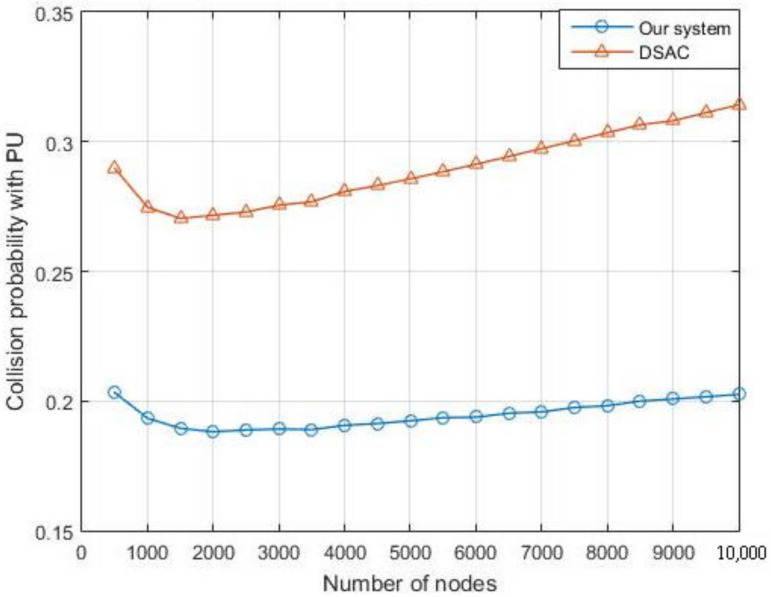
Comparison of collision with the PU transmission between our system and the DSAC.

We also compare the latency (Figure 16) and the collision probability with the PU (Figure 17) of the overall network in our system with the DSAC. The simulation shows that our system outperforms the DSAC in terms of collision probability and the delay.

## 7. Conclusions

In our study, we consider a CRSN where all nodes are provided with SS module. This network is divided into clusters and each cluster contains a number of nodes and one CH. The communication among CHs and the base station is multihop-based using a licensed channel dedicated to PU. SS is the responsibility of CHs. Upon a decision that PU is absent, CH broadcasts a request to its cluster’s nodes and its backward CHs to gather the data. In this network, a low number of clusters leads to an increase in the energy consumption due to the data transmission, while a high number of clusters increases the energy consumption due to the SS. Thus, we calculate the optimal number of clusters that saves the consumed energy within CRSN and extends the network lifespan. Latency in delivering the message and collision rate between the CH and PU transmission are also derived given the IDA adoption by the CRSN, after calculating the average number of packets retransmitted by each CH. Numerical results show that the selection of an optimal value of the cluster number and adopting IDA may considerably extend the network lifespan.

Our work makes no attempt to quantify the energy consumed during the clustering process, although clustering may consume a significant amount of energy. Therefore, as future work, we will consider all aspects related to the clustering algorithm that will be optimzed in energy consumption and simple to be implemented in CRSN. We will also derive the optimal number of clusters considering energy-harvesting-based CRSN. Moreover, it would be interesting to study the impact of number of clusters on the sustainability of the network. Another interesting perspective is to study the heterogeneous PU activity, i.e., more than one PU existing within the CRSN, on the system performance, in asymmetric links within a probabilistic network model.

## Figures and Tables

**Figure A1:**
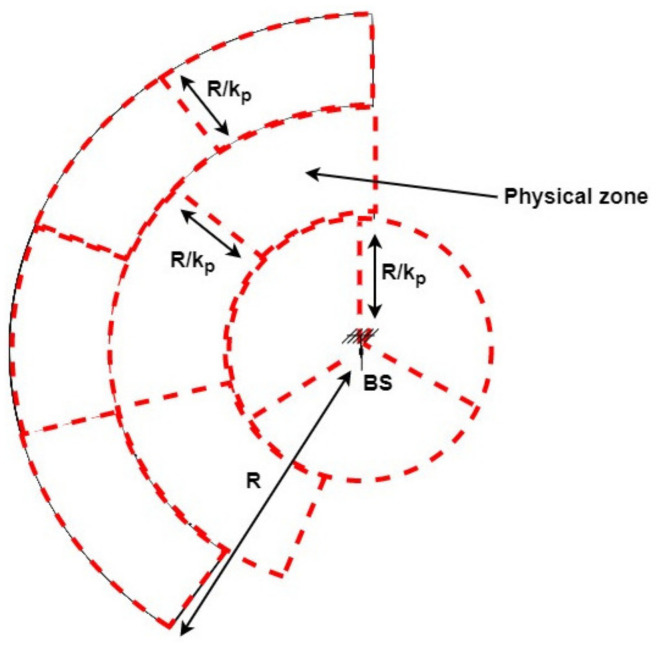
Physical zones in CRSN.

**Figure A2:**
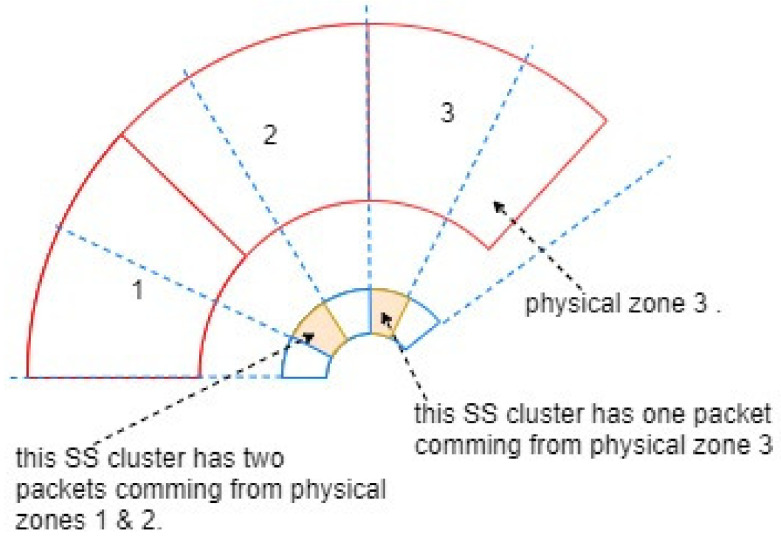
The forwarding packets coming from the physical ring *j* by the CHs in spectrum ring *i*.

## Abbreviations

The following abbreviations are used in this manuscript:

*N*	Total node numbers in the network
*M*	Number of CHs in the network
*R*	Geographical zone radius
*k*	Number of rings in network
$A_{i}$	Area of ring *i*
$m_{i}$	Number of cluster inside ring *i*
*M*	Total number of clusters in the network
$k_{p}$	Number of physical rings in the network
*p*	Number of distinct packet received by a CH
$p(H_{0})$	Probability of the PU to be idle
$p(H_{1})$	Probability of the PU to be active
$p_{fa}$	Probability of false alarm
$p_{d}$	Probability of miss detection
$p_{t}$	Probability of transmission of a CH
$N_{p_{i}}$	Average number of relayed packets by CHs in the ring *i*
$e_{r}$	Energy density at the receiver antenna
$e_{s}$	Energy consumed during SS process
$e_{0}$	Energy consumed by the node in the running mode
$e_{i}$	Average of energy consumed by a node in ring *i*
*L*	Lifespan of the network
$e_{int}$	Initial stored energy of the node
$e_{max}$	Maximum energy consumed by a node within a cycle
*D*	Density of the nodes in the network
$K_{opt}$	Optimal number of rings
$e_{opt}$	Optimal consumed energy
$P_{c}$	Collision probability with PU during overall data gathering
$P_{c_{i}}$	Probability of collision with PU in the ring *i*
$\tau_{s}$	Time slot of the PU activity Marcov model
$T_{a_{i}}$	Average time of transmission activity of CH in ring *i*
$T_{CN}$	Period of data packet transmission from one single CN to its CH
$\tau_{T_{i}}$	Average time of collecting data of a CH in ring *i* from one of its previous CHs
$V_{i}$	Average number of previous CHs in the ring i+1 of a CH in the ring *i*
$T_{P}$	Period of a single CH packet transmission
$mp_{j}$	Number of physical zones in a physical ring *j*

## Appendix A. Proof of Equation (20)

We assume that our CRSN contains *P* equal physical zones, as shown in Figure A1. Each zone contains an equal number of CHs collecting similar physical data packets. We can divide our network into $k_{p}$ physical rings, where the smallest ring is divided into three physical zones. Hence, the area of one physical zone is:

(A1) $$A_{p}=\frac{\pi }{3}{\left(\frac{R}{k_{p}}\right)}^{2}$$

The number of the physical zone *P* in the overall network is:

(A2) $$P=\frac{\pi R^{2}}{\frac{\pi }{3}{\left(\frac{R}{k_{p}}\right)}^{2}}=3k_{p}^{2}$$

where *R* is the radius of overall CRSN. Similarly to Equation (1), the area of the *i*th physical ring is:

(A3) $$P_{A_{i}}=\pi {\left(\frac{R}{k_{p}}\right)}^{2}\left(2i-1\right)$$

where $i\in [1,k_{p}]$. The number $m_{p_{j}}$ of physical zones in physical ring *j* is equal to:

(A4) $$m_{p_{j}}=\frac{P_{A_{i}}}{A_{P}}=3\left(2i-1\right)$$

As shown in Figure A2, CHs in the SS ring *i* forward packets coming from physical zones located in the physical ring *j*. Obviously, in this example, we have CHs that forward one packet and others forward two packets coming from the same physical zone. In general, in the same SS ring, we have a set of CHs that forward *n* packets and others that forward n+1 packets coming from the same physical ring. The average $av_{i,j}$ of the number of forwarding packets coming from a physical ring *j* by the CHs in SS ring *i* is calculated as:

(A5) $$av_{i,j}=\frac{1}{m_{i}}\sum_{t=1}^{m_{i}}\left(\left\lceil \frac{m_{p_{j}}}{m_{i}}t\right\rceil -\left\lfloor \frac{m_{p_{j}}}{m_{i}}\left(t-1\right)\right\rfloor \right)$$

The sum of average $av_{i}$ of the number of forwarding packets coming from all physical zones by the CHs in spectrum ring *i* is:

(A6) $$av_{i}=\sum_{j=\lceil \frac{k_{p}}{k}i\rceil }^{k_{p}}\frac{1}{m_{i}}\sum_{t=1}^{m_{i}}\left(\left\lceil \frac{m_{p_{j}}}{m_{i}}t\right\rceil -\left\lfloor \frac{m_{p_{j}}}{m_{i}}\left(t-1\right)\right\rfloor \right)$$

where $j=\lceil \frac{k_{p}}{k}i\rceil$ is the index of the physical ring containing the current SS ring *i*.

Finally, on average, a CH located in the ring *i* in our CRSN spends $\tau_{T_{i}}$ to forwarding packets toward its next CH located in the ring i+1 in the way to the BS:

(A7) $$\tau_{T_{i}}=\frac{T_{P}}{m_{i}}\sum_{j=\lceil \frac{k_{p}}{k}i\rceil }^{k_{p}}\sum_{t=1}^{m_{i}}\left(\left\lceil \frac{m_{p_{j}}}{m_{i}}t\right\rceil -\left\lfloor \frac{m_{p_{j}}}{m_{i}}\left(t-1\right)\right\rfloor \right)$$

## Appendix B. Proof of Equation (21)

Let $a=m_{p_{j}}$ and $b=m_{i}$, then:

(A8) $$\sum_{i=1}^{b}\left(\left\lceil \frac{a}{b}i\right\rceil -\left\lfloor \frac{a}{b}\left(i-1\right)\right\rfloor \right)=a+b-\left(a\wedge b\right)$$

Therefore, we have:

(A9) $$\left(a\wedge b\right)=a+b-\sum_{i=1}^{b}\left(\left\lceil \frac{a}{b}i\right\rceil -\left\lfloor \frac{a}{b}\left(i-1\right)\right\rfloor \right)$$

Let (a∧b)=c, then a=cM and b=cN with M∧N=1.

1. If a=b, then (a∧a)=a and:
  (A10) $$\begin{array}{cc} \; & a+b-\sum_{i=1}^{b}\left(\left\lceil \frac{a}{b}i\right\rceil -\left\lfloor \frac{a}{b}\left(i-1\right)\right\rfloor \right) \end{array}$$
  (A11) $$\begin{array}{cc} = & a+b-\sum_{i=1}^{b}\left(\left\lceil i\right\rceil -\left\lfloor i-1\right\rfloor \right) \end{array}$$
  (A12) $$\begin{array}{cc} = & a+b-\sum_{i=1}^{b}1=a \end{array}$$
2. If a>b, then we get:
  (A13) $$a+b-\sum_{i=1}^{b}\left(\left\lceil \frac{M}{N}i\right\rceil -\left\lfloor \frac{M}{N}\left(i-1\right)\right\rfloor \right)=b-\sum_{i=1}^{b-1}\left(\left\lceil \frac{M}{N}i\right\rceil -\left\lfloor \frac{M}{N}i\right\rfloor \right)$$
  As we can see, if $\frac{M}{N}i\in N$, then $\left\lceil \frac{M}{N}i\right\rceil -\left\lfloor \frac{M}{N}i\right\rfloor =0$
  In this case, we have i∈S={N,2N,...,(c−1)N}; then, we get:
  (A14) $$\begin{array}{cc} \; & b-\sum_{i=1}^{b-1}\left(\left\lceil \frac{M}{N}i\right\rceil -\left\lfloor \frac{M}{N}i\right\rfloor \right)\\ \; & =b-\left(\sum_{i=1}^{b-1}1-card\left(S\right)\right)\\ \; & =b-b+c=c \end{array}$$
3. If b>a, it can be proved similarly to the case of a>b.

## Appendix C. Proof of Equation (26)

According to Equation (A2), if $k_{p}=1$, that means that we have just three distinct physical zones, in this case:

(A15) $$N_{p_{i}}=\frac{1}{m_{i}}\sum_{j=1}^{1}\left(m_{p_{1}}+m_{i}-\left(m_{p_{1}}\wedge m_{i}\right)\right)$$

where, according to Equation (A4), $m_{p_{1}}=3$. We get:

(A16) $$N_{p_{i}}=\frac{1}{m_{i}}\sum_{j=1}^{1}m_{i}=1$$

which means all CHs in the overall network relay just one packet. So, let us consider the case where $k_{p}\geq 2$. Hence, to demonstrate that $N_{p_{1}}>N_{p_{i}},\;\forall i>1$, we should prove that:

(A17) $$\begin{array}{c} k_{p}^{2}-\frac{1}{m_{i}}\sum_{j=\alpha }^{k_{p}}\left(m_{p_{j}}+m_{i}-\left(m_{p_{j}}\wedge m_{i}\right)\right)>0 \end{array}$$

where $\alpha =\lceil \frac{k_{p}}{k}i\rceil$. Having:

(A18) $$\begin{array}{c} \frac{1}{m_{i}}\sum_{j=\alpha }^{k_{p}}\left(m_{p_{j}}+m_{i}\right)>\frac{1}{m_{i}}\sum_{j=\alpha }^{k_{p}}\left(m_{p_{j}}+m_{i}-\left(m_{p_{j}}\wedge m_{i}\right)\right) \end{array}$$

Inequality (A17) can be bounded by inequality (A18). We should prove:

(A19) $$k_{p}^{2}-\frac{1}{m_{i}}\sum_{j=\alpha }^{k_{p}}\left(m_{p_{j}}+m_{i}\right)>0$$

Hence:

(A20) $$\begin{array}{cc} k_{p}^{2} & -\frac{1}{m_{i}}\sum_{j=\alpha }^{k_{p}}\left(m_{p_{j}}+m_{i}\right)\\ \; & =k_{p}^{2}-\frac{1}{3(2i-1)}\sum_{j=\alpha }^{k_{p}}\left(3(2j-1)+3(2i-1)\right)\\ \; & =k_{p}^{2}-\frac{1}{\left(2i-1\right)}\sum_{j=\alpha }^{k_{p}}\left((2j-1)+(2i-1)\right)\\ \; & =k_{p}^{2}-\frac{1}{\left(2i-1\right)}\left(k_{p}^{2}+k_{p}\left(2i-1\right)+\left(1-\alpha \right)\left(2i+\alpha -2\right)\right)\\ \; & =k_{p}^{2}\left(2i-2\right)-k_{p}\left(2i-1\right)+\left(\alpha -1\right)\left(2i+\alpha -2\right) \end{array}$$

In the last equation, (α−1)(2i+α−2)≥0 since $1\leq \alpha \leq k_{p}$ and i>1. Finally, we have to prove that $k_{p}^{2}\left(2i-2\right)-k_{p}\left(2i-1\right)>0$. For this, we solve the following equation:

(A21) $$\begin{array}{c} k_{p}^{2}\left(2i-2\right)-k_{p}\left(2i-1\right)=0 \end{array}$$

Solving the equation above, we get:

$$\begin{array}{cc} k_{p} & =0,\mathrm{unacceptable}\mathrm{since}k_{p}\geq 2\\ k_{p} & =\frac{2i-1}{2i-2}<\frac{3}{2},\;\forall i>0,\mathrm{unacceptable}\mathrm{since}k_{p}\geq 2 \end{array}$$

Therefore, the last equation has the same sign as $k_{p}^{2}$. Hence, Equation (A20) is always positive. Finally, the inequality (A17) is proved.
